# The magnitude of calf morbidity and mortality and risk factors in smallholder farms across livestock production systems in central Ethiopia

**DOI:** 10.1002/vms3.877

**Published:** 2022-07-10

**Authors:** Erdachew Yitagesu, Tsegaw Fentie, Nigatu Kebede, Wendi Jackson, Woutrina Smith

**Affiliations:** ^1^ Debre Birhan Agricultural Research Center Debre Birhan Ethiopia; ^2^ University of Gondar College of Veterinary Medicine and Animal Sciences Gondar Ethiopia; ^3^ Addis Ababa University Aklilu Lema Institute of Pathobiology Addis Ababa Ethiopia; ^4^ One Health Institute School of Veterinary Medicine University of California, Davis Davis California USA

**Keywords:** calf, central Ethiopia, morbidity, mortality

## Abstract

**Background:**

Calf morbidity and mortality are major constraints in Ethiopian cattle production that severely limit available replacement stock. Calf morbidity and mortality reports in Ethiopia mostly focus on market‐oriented dairy production systems. A cross‐sectional study was undertaken in central Ethiopia with the objectives of estimating the magnitude of calf morbidity and mortality across three production systems and contributing risk factors.

**Methods:**

A cross‐sectional study was conducted at pastoral, mixed‐crop livestock, and dairy farms in central Ethiopia from February 2019 to June 2019 to collect 1‐year retrospective and cross‐sectional data on calf morbidity and mortality from smallholder farmers using a structured questionnaire.

**Results:**

A total of 293 smallholder farmers were involved in the study. Among the households interviewed, 83% of respondents encountered feed shortages in the year prior to this study. The overall annual calf morbidity prevalence and mortality rate were 6.49% (95% CI: 4.87–8.44) and 10% (95% CI: 8.28–11.93), respectively. Morbidity was higher in Dalocha and Sululta districts in mixed crop‐livestock and peri‐urban production systems, respectively. Logistic regression analysis of potential risk factors indicated that calf morbidity was associated with the calf and dam body condition score (BCS). Calves with BCS of 3 (medium) were less likely to be morbid (odds ratio [OR]: 0.20 [95% CI: 0.07–0.56]) than calves with BCS of 1 (emaciated), and calves born from dams with a body condition score of 2 (thin) were also at lower risk (OR: 0.25 [95% CI: 0.07–0.95]) than calves born from emaciated dams. The odds of calf mortality in Awash Fentale district were higher (OR: 6.19 [95% CI: 2.09–18.32]) compared to Sululta district.

**Conclusions:**

The study results revealed that the production system and management affect the magnitude of calf morbidity and mortality. We recommend improving water and feed access and resources for livestock owners to reduce calf morbidity and mortality.

## Graphical abstract

Livestock owners in the study area encountered feed and water shortages. The overall animal level estimated morbidity prevalence and mortality rates were 6.49% (95% CI: 4.87–8.44) and 10% (95% CI: 8.28–11.93) respectively. We recommend improving water and feed access and resources for livestock owners to reduce calf morbidity and mortality.

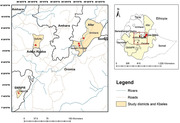

## 1 INTRODUCTION

The successful rearing of dairy calves from birth to weaning depends on a well‐managed combination of a healthy dam, a clean calving area, and early ingestion of good quality and an adequate volume colostrum. Calf morbidity and mortality are a problem in all countries where cattle are raised (Heinrichs & Radostits, 2001) but can be higher in tropical than temperate regions for a variety of reasons. Nonindigenous dairy cow breeds such as Holsteins and Jerseys are temperate animals that are most comfortable at 6–18°C (Moran, 2005, 2011). High tropical temperatures and humidity introduce specific climatic stressors that adversely affect calf and heifer feed intake, growth rates, and fertility. The tropical environment encourages the proliferation of many pathogens that can reduce calf and heifer performance through disease. Inadequate quality feed, poor husbandry practices, and reduced attention to young stock management as a result of diverse responsibilities in small‐scale mixed farming systems increase calf morbidity and mortality in tropical regions (Moran, 2011). Diarrhoea and respiratory disease are the two leading causes of mortality in dairy calves (Heinrichs & Radostits, 2001).

Calf morbidity and mortality are the two most important constraints for improving peri‐urban and urban dairy production in Ethiopia. Annual calf mortality in urban and peri‐urban dairy production systems is reported to be in the range of 15.3%–25% (Fentie et al., 2020). Similarly, 62% morbidity and 22% mortality are reported in market‐oriented smallholder dairy farms of central Ethiopia (Wudu et al., 2008). Only a small number of studies have been conducted on calf mortality and morbidity in Ethiopia, and those have focused on market‐oriented urban and peri‐urban smallholder and commercial farms (Asseged & Birhanu, 2004; Fentie et al., 2020; Ferede, 2015; Sisay & Dessie, 2017; Wudu et al., 2008). The objectives of this study were to estimate the magnitude of calf morbidity and mortality and to identify farm‐ and animal‐level risk factors associated with calf morbidity and mortality in the peri‐urban, mixed crop‐livestock and pastoral production systems in Ethiopia.

## 2 MATERIALS AND METHODS

### 2.1 Description of the study sites

**FIGURE 1:**
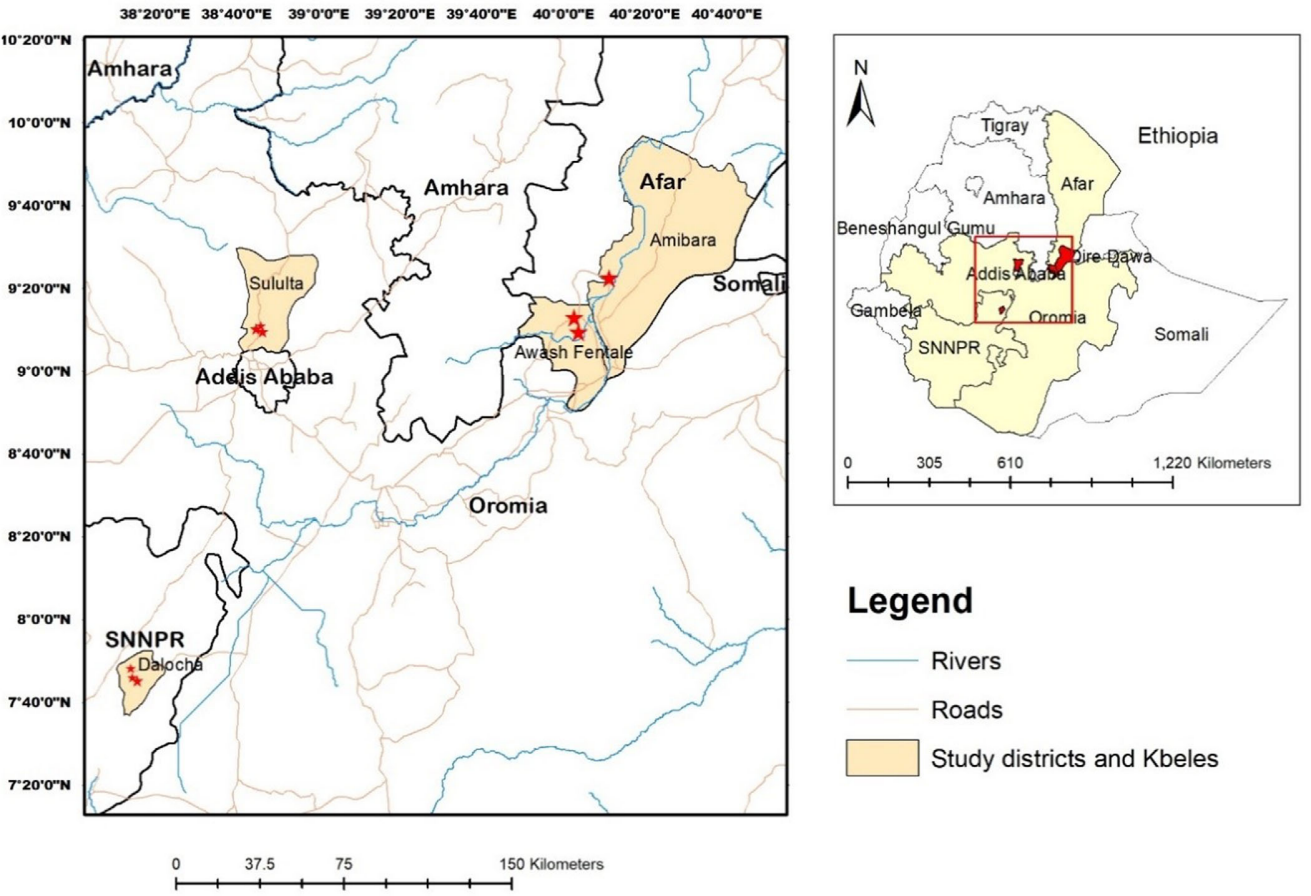
Map of Ethiopia showing the regions where the study districts and kebeles are located. Sululta is located in the Oromia region and is characterized by peri‐urban dairy production. Dalocha district is located in the Southern Nations, Nationalities, and Peoples' Region (SNNPR) and has mixed crop‐livestock farming. Awash Fentale and Amibara districts are located in the Afar region and have pastoral farming characteristics

The study was conducted across four districts in three regions of central Ethiopia. These districts included Sululta (Oromia), Dalocha (South Nations, Nationalities and Peoples), and Amibara and Awash Fentale (Afar) regions. The location of districts and each surveyed kebele (smallest administrative unit in Ethiopia) are shown in Figure 1. The study areas were selected to represent three major Ethiopian livestock production systems: mixed crop‐livestock system (Dalocha district), pastoral production system (Amibara and Awash Fentale districts), and market‐oriented peri‐urban dairy production system (Sululta district).

### 2.2 Study population and sampling method

The study population consisted of local zebu and Holstein Friesian breeds of cattle and their crosses. The target population was calves 6 months of age and younger in the four study districts in central Ethiopia: Sululta, Dalocha, Amibara, and Awash Fentale. In addition to the above four districts, Alidege kebele from Amibara district in the Afar region was included to replace Dudubi kebele in Awash Fentale because pastoral households had temporarily moved in search of feed and water for their cattle herds at the time of study enrolment.

Multistage cluster sampling was used in selecting kebeles in each district from which farms were enrolled. From Sululta and Dalocha districts, six kebeles (three from each district), two kebeles from Awash Fentale, and one substitute kebele from Amibara district were included in the study. From each kebele, smaller clustered villages (1–4 villages per kebele) that had denser cattle populations and were accessible from nearby roads were targeted. The majority of households in each village that had calves less than 6 months of age were surveyed. On a rare occasion, calves slightly older than 6 months of age were included when there was an insufficient number of calves below 6 months of age in the study kebele to enrol. The selection of villages and households was facilitated with the help of an extension veterinarian from each kebele. A household head or family member who was knowledgeable of livestock management for the selected herd/farm was interviewed to collect household and animal‐related management, calf morbidity, and mortality data.

### 2.3 Study design and sample size determination

A cross‐sectional study was conducted from February 2019 to June 2019. The study comprised a household and animal‐level questionnaire and a physical examination of each enrolled animal. Sample size determination was based on estimating proportions. However, to account for clustering at the kebele level, the baseline sample size was inflated to include an intraclass correlation coefficient. The sample size was calculated using the formula described by Bennett et al. (1991):

(1) $$n=\sqrt{\frac{pqD}{n}}=\sqrt{\frac{pqD}{cb}},$$

where *p* is an a priori estimate of the proportion, *q* = l – *p*, *D* (design effect), *n* is the required sample size, *c* is the number of clusters (kebeles), and *b* is the number of samples from each cluster (kebele). Sampling 100 animals per cluster with a prior morbidity proportion of 62% (Wudu et al., 2008) and nine clusters gave us an estimate of the standard error (1) or precision of 0.018.

(2) $$\rho =\frac{\mathrm{within}\;\mathrm{herd}\;\mathrm{variation}}{\;\mathrm{total}\;\mathrm{variation}},$$

(3) $$D=1+\left(b-1\right)\rho .$$

*Roh* (*ρ*) describes the rate of homogeneity; thus, the variability is given by (2). We use *Roh* (*ρ*) as 0.09 (Bennett et al., 1991), and taking 100 animals per cluster, *D* (design effect) equals 8.11 (3).

(4) $$c=\frac{pqD}{bSE^{2\;}}.$$

Using the equation above, an expected calf morbidity prevalence of 62% (Wudu et al., 2008) in central Ethiopia and a desired precision of 0.018%, a total of 720 animals across all nine clusters (4) (80 animals per kebele) were required to be enrolled in this study. The clusters and the total sample size were more or less equally distributed in the three main study districts (Dalocha, Sululta, Awash Fentale/Amibara). A total of 786 calves were included in the calf morbidity study.

### 2.4 Household risk factors and clinical and physical examination

Household livestock demographic data were collected using a structured questionnaire. The questionnaire was first drafted in English and then translated into the local Amharic language (Supporting Information Appendices 1–3). The questionnaire and calf clinical examination and scoring methods were pre‐tested on a small number of farms in each district with the oversight of a veterinary epidemiologist from the University of California, Davis. Local translators were used in cases where a survey respondent did not speak Amharic (for Siltie speakers in Dalocha, Afar speakers in Awash Fentale and Amibara districts and Afan Oromo speakers in Sululta districts). A physical examination was performed on every calf, and a standardized score sheet was used to assign an eye, nasal, ear, body condition and faecal score to each calf based on specific criteria developed by the University of Wisconsin and University of California, Davis. A score of 0–3 was assigned to each calf based on clinical signs related to nasal discharge, eye discharge, ear droop or head tilt, faecal score, and rectal temperature (Supporting Information Appendix 4). The calf body condition score was evaluated using a 1–5 grading system. The presence of cough, depression (not bright, alert, or responsive), separation from the group, and lack of interest in feed were also evaluated and recorded (Maier et al., 2019; McGuirk & Peek, 2014) (Supporting Information Appendix 4). Data related to the dam (parity, breed, estimated milk yield, dam body condition score [BCS], and others) were also collected along with the calf data (Supporting Information Appendices 1–3). Peri‐urban dairy farmers and some mixed crop‐livestock farmers usually measure their cows’ milk yield in litres. In most mixed crop‐livestock production and pastoral systems, farmers usually measure cows’ milk yield using locally available milking buckets and/other plastic or tin containers. This volume was converted to litres after filling the container and measuring water using a graduated jug. The dam body condition score was assigned according to criteria by Edmonson et al. (1989). Finally, each calf was categorized as healthy (no history of disease symptoms since birth reported in survey) or diseased (clinical symptoms of disease reported in survey during the past 1 year or on physical examination).

### 2.5 Data management and statistical analysis

Household and individual animal data collected during the study period were checked for completeness and entered into a pre‐formatted Microsoft Excel spreadsheet. Variables were coded and imported into STATA/SE version 17 for descriptive and inferential analyses. The calf annual morbidity prevalence per year was computed by dividing the number of calves categorized as diseased by the total number of calves sampled. Herd‐level morbidity prevalence was calculated by dividing the number of diseased herds (a herd or household reporting at least one diseased calf) by the total number of herds included in the study. Calf annual mortality was computed by dividing the number of calves that died to the total number of calves born in the study population during the previous year.

Calves were categorized into two age groups: those below or equal to three months of age were grouped as ‘younger’, and calves above three months were grouped as ‘older’ (Ferede, 2015; Wudu et al., 2008).

All calf morbidity risk factors were evaluated using univariable mixed‐effects logistic regression modelling with kebele as a random effect (melogit STATA command). Mortality data were aggregated (total number of calves that died in 1 year in each household), and a frequency weight was assigned for each household based on the total number of calves born during the year prior in each household. Then, the data were reshaped to long form using STATA ‘reshape’ syntax to be used for the logistic regression. Calf mortality risk factor analysis was performed using a mixed‐effects logistic regression district as a fixed effect and kebele as a random effect variable.

Following the univariable analysis, those variables with *p*‐value < 0.25 and not collinear with each other were fitted into a multivariable model. Multicollinearity was checked based on the variance inflation factor. If the variance inflation factor between two predictor variables was above 10, only one of them was kept in the final multivariable mixed‐effects logistic regression model (Dohoo et al., 2003). The final model was obtained by a backward stepwise elimination procedure while checking for confounding. Confounding was considered present if there was at least a 30% change in the coefficients of any of the remaining variables after removing a nonsignificant value (*p* > 0.05) from the model (Dohoo et al., 2003). For the final model fit, mixed‐effects logistic regression (melogit) stata syntax and stepwise backward selection (stepwise, pr(0.2): command before the full model and run) were performed.

The intraclass correlation (ICC) coefficient was calculated for the kebele random effect (kebele) using the ‘estat icc’ post‐estimation command of STATA. This ICC was calculated as the proportion of variances at a given cluster level (random effect variable) to the total variance (sum of 19 cluster variances and residual variance) conditional on the effect of the fixed effect predictors (Dohoo et al., 2003).

## 3 RESULTS

### 3.1 Descriptive epidemiology

In this study, 293 household farmers, pastoralists, or owners of dairy farms with at least one calf less than 6 months of age in their herd were interviewed from four districts in central Ethiopia. Seventy‐six respondents out of 293 (25.9%) reported giving supplemental feeds (commercial concentrate or locally available supplement) to their pre‐weaned calves. More than half of the farmers (128 out of 234, 54.7%) gave water to their calves only once per day, whereas 13.7% and 31.6% of farmers allowed access to water more than twice a day and twice a day, respectively. Pastoralists in Ali Dege kebele, Amibara district had access to better quality water due to a modern municipal underground water system in the village. Water from this system was used to fill livestock water troughs located throughout their clustered villages. They used a generator to fill the trough with enough water to supply their herds with water twice a day (morning and afternoon). Similarly, pastoralists residing in Awash Fentale district had sufficient access to water because of their close proximity to the Awash River. However, in Dalocha district, farmers encountered water shortages for their animals throughout the year. The farmers watered their animals at far rivers that have water when it rains. During dry periods, most of the rivers did not have water, and the farmers reduced the watering frequency of their animals to once every three days. Feed shortages in the previous year were reported by 83% (217/261) of household respondents. The approximate average daily milk yield per cow in litres reported by farmers in Sululta district was 7.4 L (95% CI: 6.5–8.2), 1.4 L (95% CI: 0.9–1.9) in Dalocha, 1.6 L (95% CI: 1.5–1.7) in Amibara, and 0.8 L in Awash Fentale (95% CI: 0.7–1.0). At the herd level, 16% of calf owners (95% CI: 12–21) reported having at least one calf develop disease between birth and the time of the interview.

At the animal level, the calf crude morbidity prevalence was 6.49% (95% CI: 4.87–8.44). The highest, 11.48% (95% CI: 7.25–17), was in Dalocha, and the lowest, 0.92% (95% CI: 0.11–3.27), was in Amibara district (Table 1).

**TABLE 1:** Calf crude morbidity prevalence by district

District	Number of calves	Diseased calves	Crude morbidity %	95% CI
Sululta	303	24	7.92	5.14–11.56
Dalocha	183	21	11.48	7.25–17
Amibara	218	2	0.92	0.11–3.27
Awash Fentale	82	4	4.89	1.34–12.02
Total	786	51	6.49	4.87–8.44

Abbreviation: CI, confidence interval.

**FIGURE 2:**
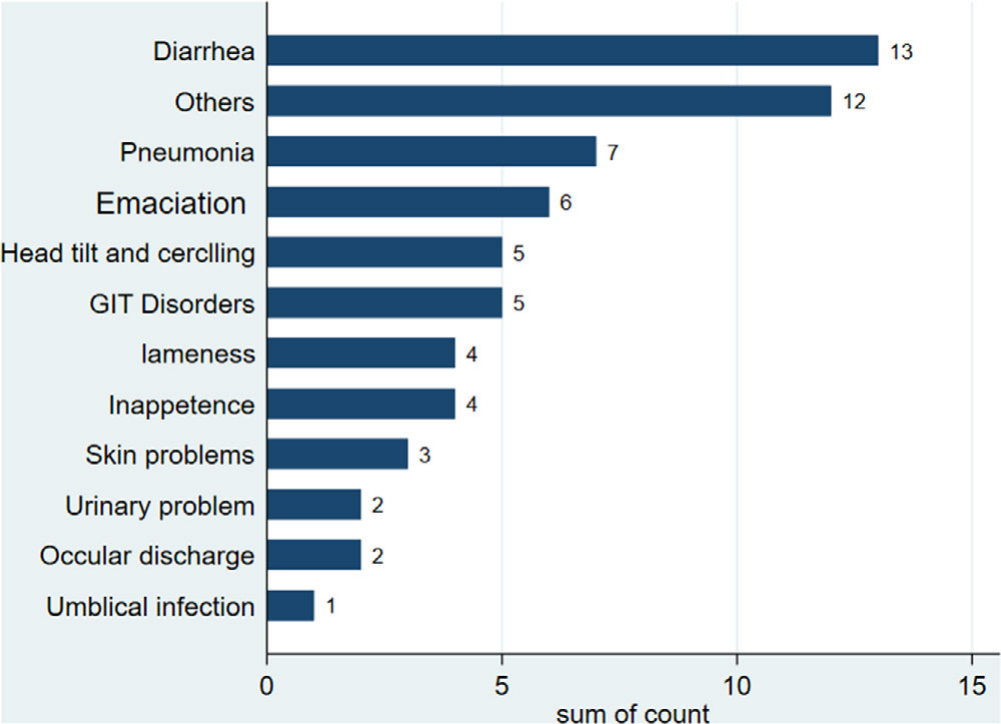
Bar graph showing the frequency of calf diseases/syndromes reported. GIT, Gasterointestinal

The most frequently reported disorders were diarrhoea and pneumonia followed by emaciation, head tilt and circling, gastrointestinal disorders (bloat, bloody faeces, straining during defecation, and other gastrointestinal‐related syndromes), inappetence, skin problems (alopecia and ecto‐parasite like flies), and others (unspecified disease syndromes such as depression, weakness, and unidentified syndromes) (Figure 2).

The overall calf crude mortality rate in the four districts was 10% (95% CI: 8.28**–**11.93). Of the 1090 total calves born across the four districts in the past 1 year, 109 (10%/year) calves had died. The highest crude mortality rate of 32.46% (95% CI: 25.15–40.47) was recorded in Awash Fentale, and the lowest was 4.14% (95% CI: 1.68–8.35) in Dalocha (Table 2).

**TABLE 2:** Calf crude mortality rate by district

District	Number of calves born	Number of calves died	Mortality rate	95% CI
Sululta	431	30	6.96	4.75–9.79
Dalocha	169	7	4.14	1.68–8.35
Amibara	336	22	6.54	4.15–9.75
Awash Fentale	154	50	32.46	25.15–40.47
Total	1090	109	10.00	8.28–11.93

Abbreviation: CI, confidence interval.

### 3.2 Risk factors for calf morbidity and mortality

Thirteen potential risk factors related to calf and dam management and production systems and their association with calf morbidity were evaluated in the univariate analysis. Of the potential risk factors related to calf and dam management, calf body condition score and dam body condition score were associated with calf disease occurrence (*p*‐value < 0.05) in the univariable mixed‐effects logistic regression model. A calf body condition score of 2 or 3 at the time of examination was protective from disease, odds ratio (OR) = 0.65 (CI: 0.31–1.37) and OR = 0.19 (CI: 0.08–0.44), respectively, compared to a calf with BCS of 1. Being born to a dam with a BCS of 2, 3, or 4 at the time of examination was also protective against disease compared to being born to dams that had a BCS of 1 at the time of examination (OR < 1). Calf sex, calf breed, dam parity group, and dam milk yield were not statistically significantly associated with calf disease occurrence (*p*‐value > 0.05) (Table 3).

**TABLE 3:** Univariable analyses of calf‐ and dam‐related risk factors for calf diseases using mixed‐effects logistic regression modelling including kebele as a random effect

Risk factors	Category	No. of sample	No. of diseased (%)	OR	95% CI	*p*‐Value
Calf sex	Female	403	28 (6.95)	1	Ref	
	Male	375	23 (6.13)	0.81	0.45–1.46	0.484
Calf breed	Local	499	27 (5.41)	1	Ref	
	Cross	287	24 (8.36)	1.60	0.66–3.85	0.299
Calf age	Younger	384	12 (3.13)	1	Ref	
	Older	402	39 (9.70)	2.13	0.99–4.56	0.053
Calf BCS	1	102	17 (16.67)	1	Ref	
	2	166	20 (12.05)	0.65	0.31–1.37	0.260
	3	403	12 (2.98)	0.19	0.08–0.44	0.000
Dam BCS	1	47	9 (19.15)	1	Ref	
	2	94	4 (4.26)	0.16	0.04–0.57	0.005
	3	510	24 (4.71)	0.23	0.09–0.56	0.001
	4	39	2 (5.13)	0.20	0.04–1.06	0.059
Dam parity	Primiparous	384	12 (3.12)	1	Ref	
	Multiparous	402	39 (9.70)	1.16	0.54–2.51	0.701
Dam daily milk yield (in litres)	0–3	478	21 (4.39)	1	Ref	
	>3–9	137	13 (9.49)	2.26	0.91–5.59	0.078
	>9–35	69	6 (8.69)	1.13	0.67–6.06	0.212

*Note*: BCS 1 = emaciated, 2 = thin, 3 = medium, 4 = heavy and 5 = fat.

Abbreviations: BSC, body condition score; CI, confidence interval; OR, crude odds ratio.

Of the management system‐ and location‐related risk factors, district and production system were significantly associated with calf disease occurrence (*p*‐value < 0.05) on univariable mixed‐effects logistic regression analysis. However, household/farm, herd size, calf rearing method, and frequency of calf access to water per day were not statistically significantly associated with calf disease (*p*‐value > 0.05) (Table 4).

**TABLE 4:** Univariable analysis of management‐ and production‐related risk factors for calf disease using mixed‐effects logistic regression modelling with kebele as a random effect

Risk factors	Category	No. of sample	No. of diseased (%)	OR	95% CI	*p*‐Value
Herd size	1–10	314	29 (9.24)	1	Ref	
	11–20	181	12 (6.63)	0.98	0.43–2.22	0.965
	21–30	70	1 (1.4)	0.22	0.03–1.72	0.148
	>30	194	6 (3.09)	0.81	0.23–2.78	0.732
Water access	Once per day	193	21 (10.89)	1	Ref	
	Twice per day	230	19 (8.26)	0.83	0.37–1.91	0.677
	>2 times/day	78	9 (11.54)	1.14	0.43–3.01	0.798
Production system	Peri‐urban	303	24 (7.92)		Ref	
	Mixed	183	21 (11.48)	1.47	0.57–3.82	0.426
	Pastoral	300	6 (2)	0.27	0.079–0.89	0.031
District	Sululta	303	24 (7.92)	1	Ref	
	Dalocha	183	21 (11.47)	1.48	0.63–3.47	0.373
	Amibara	218	2 (0.92)	0.10	0.02–0.54	0.007
	Awash Fentale	82	4 (4.89)	0.58	0.16–2.09	0.401
Calf rearing	Restricted suckling	739	47 (6.36)	1	Ref	
	Artificial rearing	50	4 (8)	1.23	0.38–3.95	0.730

Abbreviations: CI, confidence interval; OR, crude odds ratio; Ref, reference.

The multivariable model was run using variables that had a *p*‐value < 0.25 on univariate mixed‐effects analysis to obtain adjusted OR. Only statistically significant predictors and confounders were included in the final model. The final multivariate model was statistically fit with a log likelihood value of −124.26 and a *p*‐value of 0.0062. The calf BCS and dam body condition score were included in the final model and were statistically significantly associated with estimated calf morbidity. Calves with a body condition score of 3 at the time of evaluation had a lower risk of morbidity than calves with a body condition score of 1 (OR: 0.25 [95% CI: 0.09–0.68]). However, calves with a body condition score of 2 were not significantly associated with risk of morbidity compared to calves with a body condition score of 1 (OR: 0.72 [95% CI: 0.29–1.77]) in the multivariable model. Calves with a dam with body condition score of 2 at the time of evaluation were statistically significantly associated with a lower risk of morbidity than calves with a dam with BCS of 1 (OR: 0.25 [95% CI: 0.07–0.95]) (Table 5).

**TABLE 5:** Final multivariable model of risk factors related to calf diseases at the animal level (*n* = 560) using mixed‐effects logistic regression modelling with kebele as a random effect

Risk factor	Category	Odds ratio	SE	95% CI	*p*‐Value
Calf BCS	1	1		Ref	
	2	0.72	0.33	0.29–1.77	0.479
	3	0.25	0.13	0.09–0.68	0.006
Dam BCS	1	1		Ref	
	2	0.25	0.17	0.07–0.95	0.042
	3	0.45	0.23	0.17–1.23	0.116
	4	0.49	0.45	0.08–2.93	0.439
Intrakebele variance		0.32	0.209	0.05–2.14	
ICC kebele		0.09	0.08	0.01–0.39	

*Note*: BCS 1 = emaciated, 2 = thin, 3 = medium, 4 = heavy and 5 = fat.

Abbreviations: CI, confidence interval; ICC, intracluster correlation coefficient; Ref, reference; SE, Standard error; BSC, Body condition score.

The intrakebele variance was estimated as 0.32, resulting in an ICC of 0.09 using the estat icc post‐estimation command of Stata (Table 5). Using formula 3, the design effect (*D*) was 8.91. The large ICC indicates similarity in the risk of exposure to morbidity and mortality for calves within a given kebele.

Calf mortality risk factor analysis was performed using mixed‐effects logistic regression with district as a fixed effect and kebele as a random effect. The OR of calf mortality in Awash Fentale was 6.19 times higher than that in Sululta. However, a calf reared in Dalocha and Amibara districts did not have a significant mortality risk effect compared to Sululta (OR = 0.48 [95% CI: 0.14–1.65]; 0.86 [95% CI: 0.23–3.25]) (Table 6). Using formula 3, the design effect (*D*) was 8.92.

**TABLE 6:** Univariable analysis of calf mortality mixed effect logistic regression model with district as fixed effect and kebele as random effect

District	OR	SE	95% CI	*p*‐Value
Sululta	1		Ref	
Dalocha	0.48	0.30	0.14–1.65	0.245
Amibara	0.86	0.58	0.22–3.25	0.819
Awash Fentale	6.19	3.43	2.09–18.32	0.001
Intrakebele variance	0.28	0.22	0.06–1.26	
ICC kebele	0.08	0.06	0.02–0.28	

Abbreviations: CI, confidence interval; ICC, intraclass correlation; OR, odds ratio; SE, standard error.

## 4 DISCUSSION

There are inherent limitations in comparing morbidity and mortality data across studies. These limitations have been described previously by Ferede (2015) and Wudu et al. (2008) and include decreased reliability in morbidity compared to mortality data in dairy systems, since mortality is a significant event that is more easily remembered compared to illness. This stems from the potential for recall bias when producers are asked to describe clinical signs associated with sick animals when each producer may recall symptoms differently. It can also be difficult for producers to recall every illness event (Heinrichs & Radostits, 2001). Variations in morbidity and mortality can be explained by many calf‐ and herd‐level risk factors, case definition, age of the calves, study design, sample size, and agro ecology (Windeyer et al., 2014), which vary across studies. Potential reasons for the variation found in calf morbidity and mortality rate in the present study may include differences in study design and target population. Our estimates for crude morbidity and mortality were low because the target age group for enrolment was restricted primarily to calves 6 months of age and younger. Therefore, it is difficult to compare unadjusted prevalence in terms of animal time at risk. Furthermore, seasonal variation and recurrent drought, particularly in pastoral areas, affect the magnitude of calf morbidity and mortality in different study years (Fentie et al., 2020; Waltner‐Toews et al., 1986). During our study period, there was no drought or major disease outbreak occurrence in the study districts. A strength of this study was that the questionnaire, sampling, and enrolment criteria were consistent across three different production systems.

In the present study, the estimated annual crude mortality rate across the four districts (Sululta, Dalocha, Awash Fentale, and Amibara) was 10%. The calf crude mortality rate (10%) reported in this study agreed with previous research reports: 9.7% (Haile‐Mariam et al., 1993), 9.3% (Megersa et al., 2009) and 11.6% (Romha, 2014) in different livestock production systems of Ethiopia. Similarly, Otte and Chilonda (2002) reported 12.4% and 15% mortality rates in female and male calves, respectively, in small‐scale dairy systems and 8.1% and 6.4% in female and male calves, respectively, in large‐scale dairy production systems in sub‐Saharan African countries.

Calf morbidity and mortality in the present study were considerably lower than most of the previous reports in different parts of Ethiopia (Asseged & Birhanu, 2004; Fentie et al., 2020; Ferede, 2015; Sisay & Dessie, 2017; Wudu et al., 2008). Wudu et al. (2008) reported 62% and 22% crude morbidity and mortality rates, respectively. Ferede (2015) reported 47.3% crude morbidity and a 17.9% crude calf mortality rate, and Sisay and Dessie (2017) reported 21.45% mortality. Asseged and Birhanu (2004) reported a 20% mortality rate. Fentie et al. (2020) reported calf mortality in the range of 9.4%–14% in mixed crop‐livestock production, 15%–25% in urban and peri‐urban dairy production, and 26%–29.2% in pastoral production systems where there was severe drought in this study period. Dagne et al. (2018) reported the highest calf mortality rate of 58.37% in Sululta district. The low morbidity and mortality in this study could be due to calf age restriction (<6 months), and the magnitude differences could be a result of variations in livestock management, time of study, and sample size.

The primary causes of morbidity in our study area were diarrhoea and pneumonia, which is in agreement with previous reports (Asmare & Kiros, 2016; Dagne et al., 2018; Fentie et al., 2020; Ferede, 2015; Megersa et al., 2009; Wudu et al., 2008).

Multivariable risk factor analysis of the explanatory predictor variables for calf morbidity in this study found that calf and dam body condition scores at the time of evaluation were statistically significantly associated with morbidity. Calves with a higher body condition score of 3 were at lower risk of morbidity (OR: 0.25 [95% CI: 0.09–0.68]) than calves with a body condition score of 1. However, this result cannot be conclusive, as a lower calf body condition can reversibly be due to calf sickness. Similarly, calves born from a dam with a BCS of 2 at the time of evaluation were statistically significantly associated with a lower risk of morbidity than calves born from a dam with a BCS of 1. In households with inadequate feed and poor feed quality, calves and dams may have lower body condition scores that may increase their risk for disease.

There was a significant difference in mortality rate between Awash Fentale (pastoral production) and Sululta (peri‐urban production), with an OR of 6.19 higher mortality risk in Awash Fentale. The mortality variation can be explained by the difference in environment or production system. Calves in the pastoral production system encounter greater health risks stemming from a harsh environment, with recurrent drought and poor management. This finding is consistent with results from previous studies (Fentie, 2016; Otte & Chilonda, 2002). Exceptionally, the mortality rate in Amibara district is lower than that of its neighbouring pastoral district, Awash Fentale. The lower mortality in Amibara is most likely due to better water access in the area and better suitability for cattle rearing. Pastoralists in Ali Dege kebele, Amibara district, have clean underground water in their village, and their herds have access to water twice a day, once in the morning and once in the afternoon. In addition, they are more dependent on cattle production, and they have a larger area of quality grazing land for their cattle. However, cattle production in the Doho and Kebene kebele of Awash Fentale district does not have adequate water resources in proximity to their village and travel long distances to get feed for their livestock. The morbidity prevalence in Ali Dege, Amibara district was also lower (1%) than that in Dalocha district (11%). The higher morbidity prevalence in Dalocha district can probably be due to scarcity of water and feed in the area.

## 5 INTRACLASS CORRELATION COEFFICIENT

The intrakebele variance of 0.32, ICC value of 0.09 (9% variability in calf morbidity between individual calves was due to difference in kebeles), and design effect (*D*) 8.91 obtained in our multivariable mixed effect logistic regression model for morbidity was relatively large and indicative of increased variation in morbidity between kebeles. Similarly, the intrakebele variance of 0.28, ICC value of 0.08 (8% variability in calf mortality between individual calves was due to difference in kebeles), and design effect (*D*) 8.92 obtained from mortality mixed effect logistic regression was also relatively large, indicating the presence of variation in mortality between kebeles.

## 6 CONCLUSIONS AND RECOMMENDATIONS

Findings from this study shed light on how a scarcity of water and feed resources influences the magnitude of calf morbidity and mortality rates in different livestock production settings in Ethiopia. Villages that have better clean water access and good grazing land in the pastoral area reported lower calf morbidity and mortality occurrences. Diarrhoea and cough were the most common calf disease symptoms reported during the household interview. Risk factor analysis for calf morbidity revealed that dam and calf body condition scores at the time of evaluation were the main factors associated with a calf experiencing disease, whereas calf mortality rate was associated with the district where calves were raised. We recommend improving water and feed access and resources, particularly in pastoral and mixed crop‐livestock production areas. Providing proper feed along with frequent access to water for calves in their early life through extension education and training directed at farmers is critical for reducing calf morbidity and mortality.

## CONFLICT OF INTEREST

The authors declare no conflict of interest.

## ETHICS STATEMENT

The authors confirm that the ethical policies of the journal, as noted on the journal's author guidelines page, have been adhered to. The University of California, Davis, Institutional Animal Care and Use Committee (IACUC) approved this research (protocol # 19666). Additional approval was granted by University of Gondar and Addis Ababa University, Ethiopia. All efforts were made to minimize animal suffering during physical examination.

## AUTHOR CONTRIBUTIONS

*Conceptualization, data curation, formal analysis, investigation, methodology, resources, software, validation, visualization, writing—original draft, and writing—review and editing*: Erdachew Yitagesu Yitagesu. *Data curation, funding acquisition, resources, supervision, and writing—original draft*: Tsegaw Fentie. *Conceptualization, investigation, project administration, and supervision*: Nigatu Kebede. *Investigation, methodology, project administration, supervision, writing—original draft, and writing—review and editing*: Woutrina Smith: *Funding acquisition, investigation, resources, and supervision*: Wendi Jackson.

## Supporting information

Appendix 1 Household risk factor identification forms
Appendix 2 Calf enrolment forms
Appendix 3 Physical exam forms

Appendix 4 Field Sample Collection Reference Documents for Calf Enrolment

## Data Availability

The data that support the findings of this study are available from the corresponding author upon reasonable request.
