# Supplementary material for: The magnitude of calf morbidity and mortality and risk factors in smallholder farms across livestock production systems in central Ethiopia
Source: Vet Med Sci. 2022 Jul 10;8(5):2157–66. doi: 10.1002/vms3.877 (PMC9514487; doi:10.1002/vms3.877)
Supplement: Supplementary file 1 — Appendix 1 Household risk factor identification forms Appendix 2 Calf enrolment forms Appendix 3 Physical exam forms [file VMS3-8-2157-s002.docx]

Appendix 1 Household risk factor identification forms

| 1) Household ID ASSIGNED (Per ID protocol, i.e., G-F001) |  |
| --- | --- |
| 2) Interview performed by (full name): |  |
| 3) Date (Western Calendar): | DD: MM: YYYY: |

**Owner information**

| 4) Livestock Owner name (optional) | |  | | |  | |  |
| --- | --- | --- | --- | --- | --- | --- | --- |
| 5) Primary telephone contact number | |  | | |  | |  |
| 6) gender | | male | | | female | | |
| 7) Owner educational status | | 0=none(preschool) 1=primary 2=secondary 3=higher 4=don’t know 5= no response | | | | | |
| 8) District/Woreda | Sululta | | | Dalocha | | | Awash fentale |
| 9) Kebele | 01 Sululta  Wale lube  Nano mana Abichu | | | Dalocha tilkisa  Jagena lesho  Getie kutiyo | | | Dudubi  Kebena  Doho |
| Village |  | | | | Altitude | |  |
| 10) GPS coordinates of farm: (WGS 1984) | | | Latitude | |  | Longitude | |
| 11) Predominant Production System | | Peri-Urban Dairy | | | Mixed Crop Livestock | | Pastoral |

**Farm information and facilities**

1. How many animals on average have you have during the last year of the following types:

| Age group | Adult females (older than 2 years old) | Young females (6 months - 2 years old) | Female calves (less than 6 months old) | Adult males (older than 6 months old) | Male calves (less than 6 months old) |
| --- | --- | --- | --- | --- | --- |
| Number |  |  |  |  |  |

Out of all the cows that were inseminated and pregnant in the last 12 months, how many…?

| Reproductive problems yes/no) | Dams aborted | stillbirth | Weak calf | Dystocia | Uterine or vaginal prolapse | NR |
| --- | --- | --- | --- | --- | --- | --- |
| Number |  |  |  |  |  |  |
| Reproductive problems | Retained foetal membrane (>12hrs) | Uterine infection | Repeat breeding(cow>3x) | Offspring born alive | Prolonged calving (cow >18months |  |
| Number |  |  |  |  |  |  |

1. Of the offspring born alive, how many...?

| Died within 24 hours | Died (2 – 7 days) | Died (8 – 30 days) | Died (31 – 90 days) | Died after 90 days | Is still alive | Don’t know | NR |
| --- | --- | --- | --- | --- | --- | --- | --- |
|  |  |  |  |  |  |  |  |

1. Where do births usually happen?

| Separate birth area | Same area as the herd | Don’t know | NR |
| --- | --- | --- | --- |
|  |  |  |  |

What do you do with placentas, fetal membranes and aborted fetuses?

| Bury | Incinerate | Throw it into river | Throw it into latrine | Feed it to the dogs | Other, Specify | NR |
| --- | --- | --- | --- | --- | --- | --- |
|  |  |  |  |  |  |  |

1. Do you separate sick animals from the rest of the herd?

|  | Yes | No | Don’t know | NR |
| --- | --- | --- | --- | --- |
|  |  |  |  |  |

1. How do you dispose of died animals? Please, specify by species.

| feed it to dogs | renderer | Incineration | on-site composting | on-site burial | other | Don’t know | NR |
| --- | --- | --- | --- | --- | --- | --- | --- |
|  |  |  |  |  |  |  |  |

1. How often do you clean the floors/ground surface where young stock are kept?

| At least once a day | Several days per week | Once a week | Several times per month | Several times per year | Don’t know | NR |
| --- | --- | --- | --- | --- | --- | --- |
|  |  |  |  |  |  |  |

**HOUSING**

1. Do animals of all ages share the same housing areas?

| Yes | If yes, what ages are housed together? | No | Not housed | NR |
| --- | --- | --- | --- | --- |
|  |  |  |  |  |

1. Type of facilities where animals are housed:

| Animal barn/shed with roof and at least one window (not including door) that opens and closes |  |
| --- | --- |
| Animal barn/shed with roof with no windows (not including door), or windows that do not open |  |
| Pen without roof |  |
| Human dwelling |  |
| Left loose |  |
| Tied outdoors |  |
| Other, specify |  |
| NR |  |

1. Where the calves are kept, what is the primary flooring surface?

| Soil | Concrete/Bricks | Rubber mats | Elevated wood slats with spaces | Wood without slats | Other, specify | NR |
| --- | --- | --- | --- | --- | --- | --- |
|  |  |  |  |  |  |  |

Type of bedding on top of the primary calf flooring surface

| Straw | Sawdust/Wood | Other, specify | Bedding is not used | NR |
| --- | --- | --- | --- | --- |
|  |  |  |  |  |

- 1. Type of stalls for cattle:

Tie-stall Free-stall other, specify: NR

**BREEDING & REPRODUCTION**

1. Type of breeding performed in the farm:

Cattle: Natural breeding Artificial Insemination (AI) NR If Artificial Insemination (AI),

What is the source of AI semen? _______________________________________________________

How many services are typically required for successful breeding? __________________

How many cows got pregnant in the last year (regardless of later pregnancy loss?

Do you currently use any clean-up bull? Yes No NR

1. Are the dams separated from the herd when she is close-up? Yes No NR
2. During births, how do you decide when it is time to help the dam to deliver the calf________________________________________________________________________
3. How often do aborted animals have hair (indication of abortion in late gestation)?

Always Never

Calves 1 2 3 4 5 NR

1. Of the animals that gave birth in the last 12 months, how many had retained placenta (12 hours after birth still had not expelled the placenta)? Cows

**WATER & NUTRITION**

1. What is the source of the water you give to the animals? Check all that apply.

___ Piped water ___ Tube well or borehole ___ Protected well ___ Unprotected well

___ Protected spring ___ Unprotected spring ___ Rainwater ___ Tanker truck ___ Cart with small tank

___ Surface water (river, dam, lake, ponds, stream, canal, irrigation channel)

1. Do animals graze for part of the day during the entire year?

Yes No, it varies by season NR

Appendix 2 Calf enrolment forms

| 1) YOUNG STOCK ID ASSIGNED (Per ID protocol) | | |  | | | | |  |  |
| --- | --- | --- | --- | --- | --- | --- | --- | --- | --- |
| 2) Age | | (days) | (Weeks) | | (months) | | | Don’t know | |
| 3) Sex (circle one) | | Male | Female | | |  |  |  |  |
| 4) Predominant Breed (circle one) | | Local | Crossbred (%) | | | | Don’t know | |  |
| 5) Name of Breed | |  | | | | Don’t know | | |  |

**ANIMAL HISTORY**

For the following questions, circle one option.

| 6) Sire | Artificial Insemination | Live Cover | | Don’t know |
| --- | --- | --- | --- | --- |
| 7) Time of Birth | Day | Night | | Don’t know |
| 8) Birth witnessed | Yes | No | | Don’t know |
| 9) Birth location | In barn | outside | Other | Don’t know |
| 10) Type of delivery | normal | assisted | | Don’t know |
| 11) Colostrum fed within 24 hours? | Yes | No | | Don’t know |
| 12) If answer to question 11 is no, was this because | |  | |  |
| Calf alert but unable to suck | Calf depressed, unable to suck | Other | | Don’t know |
| 13) Navel dipped | Yes | No | | Don’t know |
| 14) Did animal have continuous access to dam for the first 24 hours of life? | Yes | No | | Don’t know |
| 15) Separate housing from herd? | Yes | No | | Don’t know |
| 16) Animal housed with dam? | Yes | No | | Don’t know |
| 17) Animal housed with other young stock? | Yes | No | | Don’t know |

| 18) Any vaccinations given to animal since birth? | | Yes | | | No | | Don’t know | |  |
| --- | --- | --- | --- | --- | --- | --- | --- | --- | --- |
| 19) If yes, what type of vaccines? | |  | | |  | |  | |  |
| 20) Any medications given to animal since birth? | | Yes | | | No | | Don’t know | |  |
| 21) If yes to question 20, what type of medications? Mark from the following below | | | | | | | | |  |
| Dewormers | | Yes | | | No | | Don’t know | |  |
| Vitamins | | Yes | | | No | | Don’t know | |  |
| Oral electrolytes | | Yes | | | No | | Don’t know | |  |
| Other, specify | | Yes | | | No | | Don’t know | |  |
| 22) Has this animal shown any signs of illness since birth? | | | Yes | | | No | | Don’t know | |
| 23) If yes to question 22, mark what symptoms of illness has been observed | | | | | | | | |  |
|  | Ye | | | No | | | Don’t know | |  |
| What? describe |  | | | | | | | |  |

**ANIMAL FEEDING HISTORY**

| 24) Is animal fed milk? | Yes, MILK | | | | | Yes, MILK REPLACER | | Not fed Milk or Milk replacer | | Don’t know |
| --- | --- | --- | --- | --- | --- | --- | --- | --- | --- | --- |
| If animal is fed either milk or milk replacer, answer the following | | | | | | | | | |  |
| 25) How often is animal fed milk/milk replacer? | | | Once a day | | | Twice a day | | More than twice a day | | Don’t know |
| 26) At each feeding, how much milk is fed? | | | | | | (Liters) | | | | Don’t know |
| 27) Is animal fed supplemental food? | | | | | Yes | | | No | | Don’t know |
| If yes to supplemental food | | | | | | | | | |  |
| 28) How often is fed supplemental food | | Once a day | | | Twice a day | | | More than twice a day | | Don’t know |
| 29) Is animal given water? | | | | | Yes | | | No | | Don’t know |
|  | | | | | If yes to water | | |  | |  |
| 30) How often is the animal given water? | | Once a day | | | Twice a day | | | More than twice a day | | Don’t know |
| 31) Does animal have its own water trough? | | | | | | Yes | | No | | Don’t |
| 32) Is the animal weaned? | | | | Yes | | | No | | Don’t know | |
|  | | | | If yes to eaned | | |  | |  | |
| 33) What age was animal weaned at? | | | |  | | | (months) | | Don’t know | |

**ANIMAL ENVIRONMENT**

Inspect animal housing place. Look at where this animal is housed and answer the following questions.

| 34) Is animal housed on bedding? | Yes | No | Don’t know |
| --- | --- | --- | --- |
| 35) Does the area where the animal lies down look clean and dry? | Yes | No | Don’t know |
| 36) If all the animals housed together were going to stand up at the same time, would they have enough room to do so and turn around without touching each other? | Yes | No | Don’t know |

**DAM HISTORY**

For the following questions, circle one option

| 37) Age of dam at parturition |  | (years) | Don’t know |
| --- | --- | --- | --- |
| 38) Dam gestation number |  |  | Don’t know |
| 39) Were any vaccines given to the dam within 2 months of delivery | Yes | No | Don’t know |
| 40) If yes to vaccines, specify type |  |  |  |

| 41) Is dam alive? | Yes | | No | Don’t know |
| --- | --- | --- | --- | --- |
| If yes, answer the following | | |  |  |
| 42) Dam Body Condition Score (cows/sheep/goats use scale 1 -5; camels use scale 0-5) | | |  | Don’t know |
| 43) Dam milk production (include all milk, i.e., milk given to calf and milk sold /human consumption) | | | (Liters/day)  _____________ | Don’t know |
| 43) Is dam mothering instinct adequate? | | Yes | No | Don’t know |
| Is dam have any reproductive health problem before? | | Yes | No |  |
| If yes to the above what kind of reproductive health problem? (RB, extended parturition, abortion, still birth, dystocia, uterine infection, RFM, | |  | | |

| If no to question #43, answer the following |  |  |  |
| --- | --- | --- | --- |
| 44) Did the dam die within the first 2 weeks after parturition? | Yes | No | Don’t know |

Post - weaning addendum **–** only fill in this section if the animal has already been weaned

| 45) Grazing (hours of grazing) |  |
| --- | --- |
| 46) Legumes (Alfalfa, Sesbania, Leucaena) mixed with roughage/grazing. Specify type and quantity |  |
| 47) Concentrates, type and quantity |  |
| 48) Hay, type and quantity |  |
| 49) Urea treated straw, quantity |  |
| 50) Mineral blocks |  |
| 51) Others, type and quantity |  |
| 52) Amount of hours animals have access to water (1 time/day, 2 times/day, 3 or more times/day) |  |

Appendix 3 Physical exam forms

Before restraining the animal, make observations about general attitude.

| 1) Attitude (circle one) | Bright/Alert | Depressed | Lethargic | Comatose |
| --- | --- | --- | --- | --- |
| 2) General notes if necessary (emaciation, in pain, etc.) | | |  |  |
|  | | |  |  |

| Key to temperature score  37.7 - 38.2 = 0  38.3 - 38.8 = 1  38.9 - 39.3 = 2  >39.3 = 3 |
| --- |

| 3) Rectal  Temperature |  | Celsius |
| --- | --- | --- |
|  | | |

Once you are able to restrain the animal, record the following:

ore – USE SCORING CARD:

| Temperature  Score (^o^C)  (0-3) | Cough  Score  (0-3) | Nasal Discharge  Score  (0-3) | Eye Score  (0-3) | Ear Scoe    (0-3) | Increased Respiratory effort?  0 – no 1 - yes |
| --- | --- | --- | --- | --- | --- |

Thoracic Auscultation:

| 3) Respiratory Rate |  | Breaths per minute | |
| --- | --- | --- | --- |
| Are wheezes present? | Yes | No | Don’t know |
| Are crackles present? | Yes | No | Don’t know |
| Are lung sounds muffled? | Yes | No | Don’t know |
| Is animal coughing? | Yes | No | Don’t know |
| 4) Heart Rate |  | Beats per minute |  |
| Are there any abnormal heart sounds? | Yes | No | Don’t know |
| 5) Notes on Thoracic Auscultation |  |  |  |

Abdominal Auscultation:

| 6) Ruminal Contractions | | |  | | | Number per minute | | | |
| --- | --- | --- | --- | --- | --- | --- | --- | --- | --- |
| Are peristaltic sounds increased? | | | Yes | | | No | | | Don’t know |
| Are there any ping sounds? | | | Yes | | | No | | | Don’t know |
| 7) Notes on Abdominal Auscultation | | |  | | |  | | |  |
| 8) Body Condition Score (1-5) | | | |  | |  | | |  |
| 9) Umbilicus Examination (Use thumb as reference) | |  | | Normal | | Abnormal | | | Don’t know |
| 10) Notes on umbilicus exam | | | |  | |  | | |  |
| 11) Limb Joints Examination | |  | | Normal | | Abnormal  (Enlarged, painful, fluid accumulation) | | | Don’t know |
| 12) Notes on joints exam | | | |  | |  | | |  |
| NORMAL | Pasty, normal color | | | | Loose | | Watery | | |
| 0 | 1 | | | | 2 | | 3 | | |
| 14) Blood in stool? | Yes | | | | No | | | Don’t know | |
| 15) Notes on fecal exam | | | | |  | | |  | |
