# Supplementary material for: The magnitude of calf morbidity and mortality and risk factors in smallholder farms across livestock production systems in central Ethiopia
Source: Vet Med Sci. 2022 Jul 10;8(5):2157–66. doi: 10.1002/vms3.877 (PMC9514487; doi:10.1002/vms3.877)

Appendix
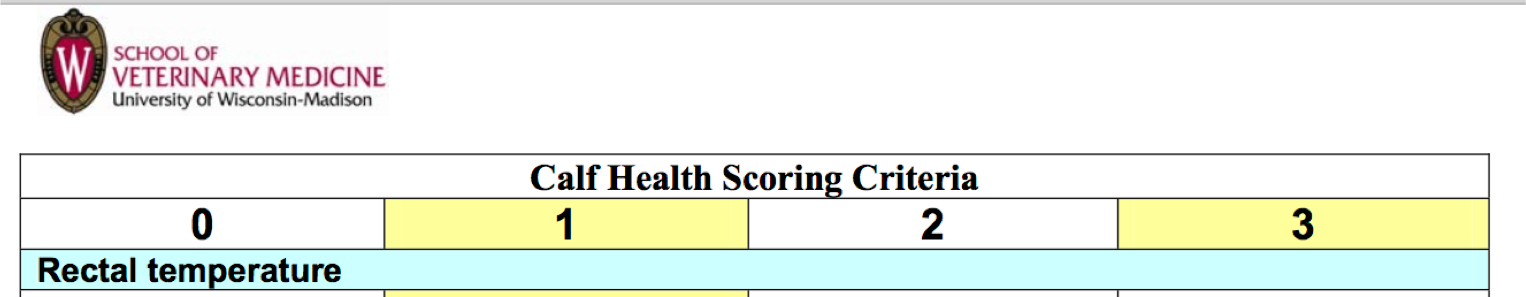
 4 Field Sample Collection Reference Documents for Calf Enrollment

**37.7-38.2 C 38.3-38.8 C 38.9-39.3 >39.3 C**


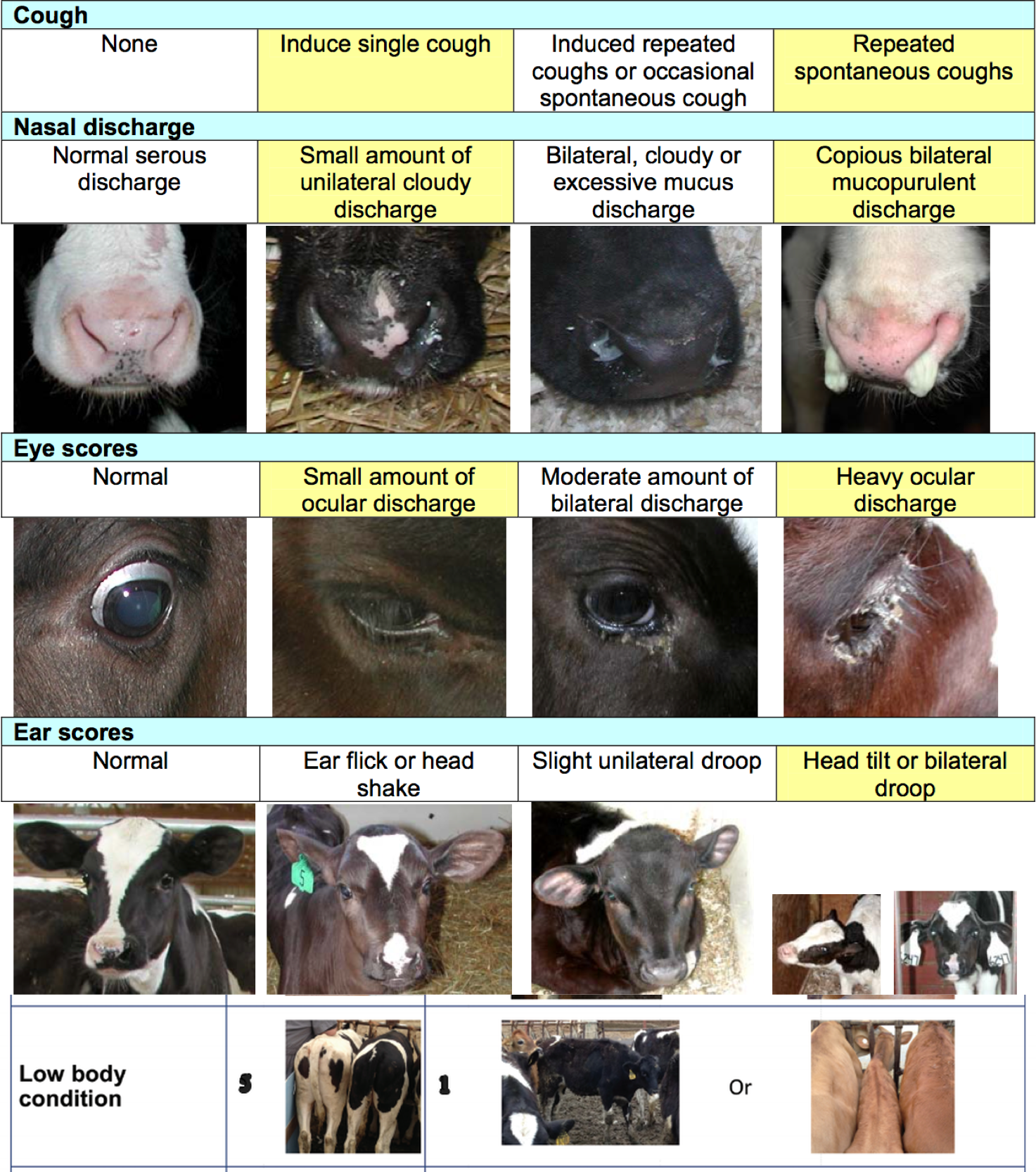


BODY CONDITION SCORE 1-5 for cows


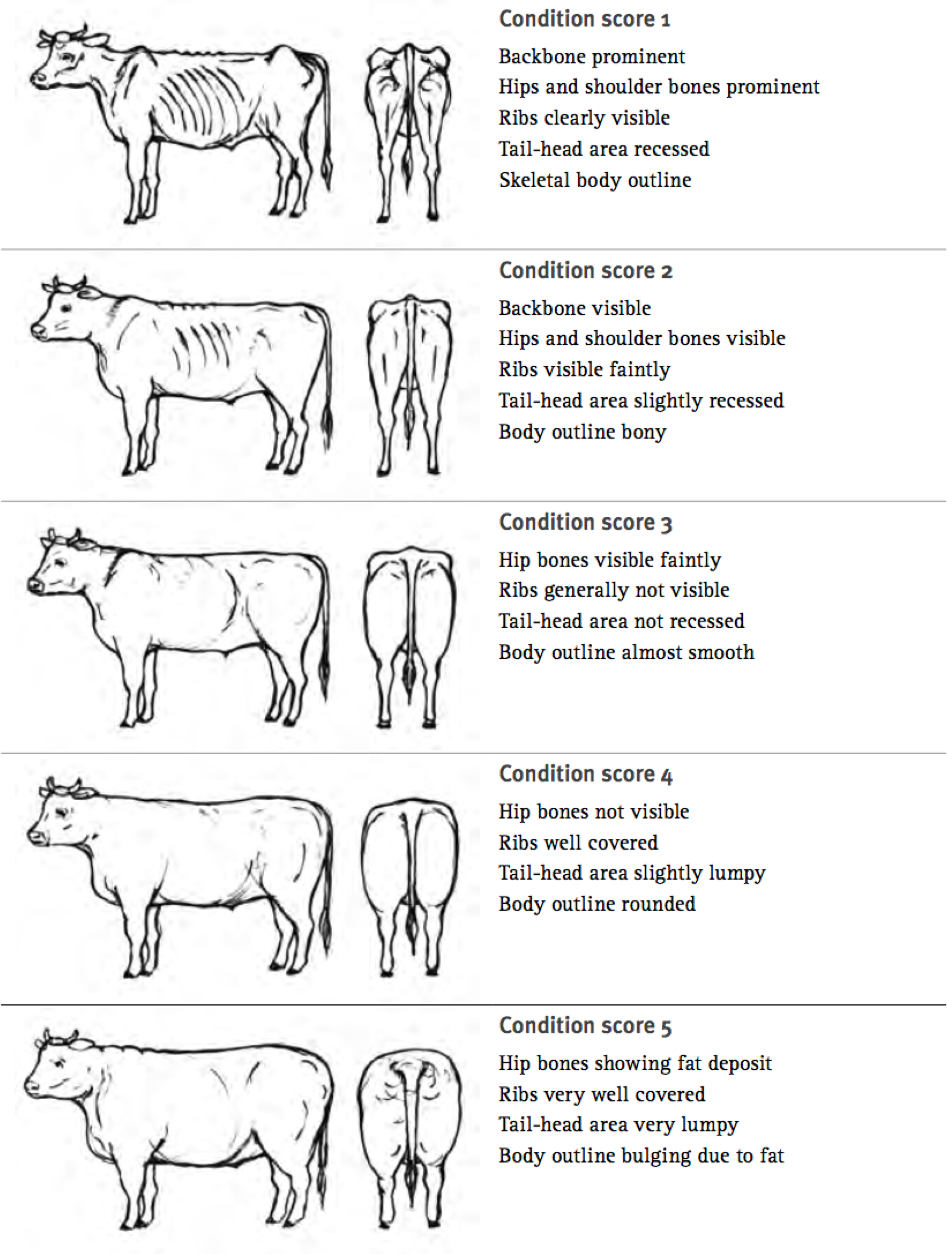


FECAL SCORE

| **0** | **1** | **2** | **3** |
| --- | --- | --- | --- |
| NORMAL | Pasty, normal color | Loose | Watery |


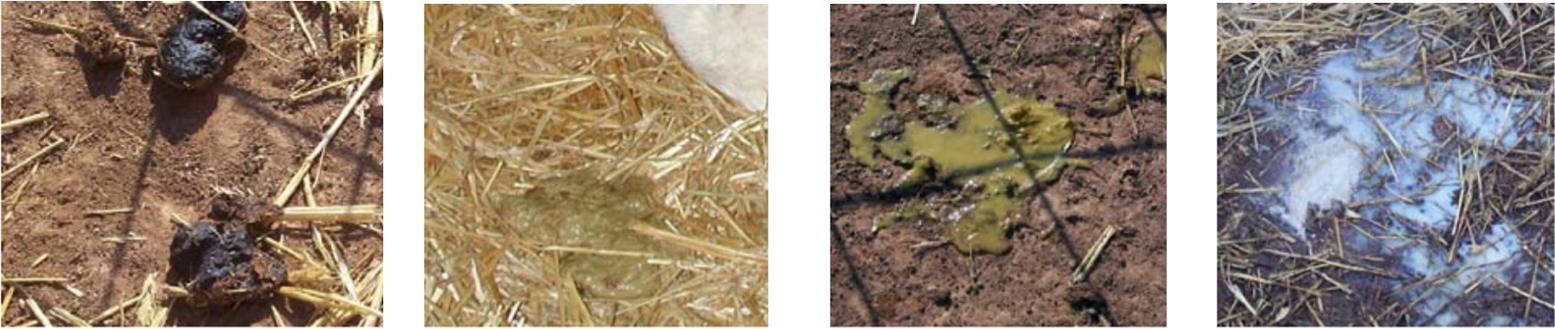


USING TEETH TO ESTIMATE AGE


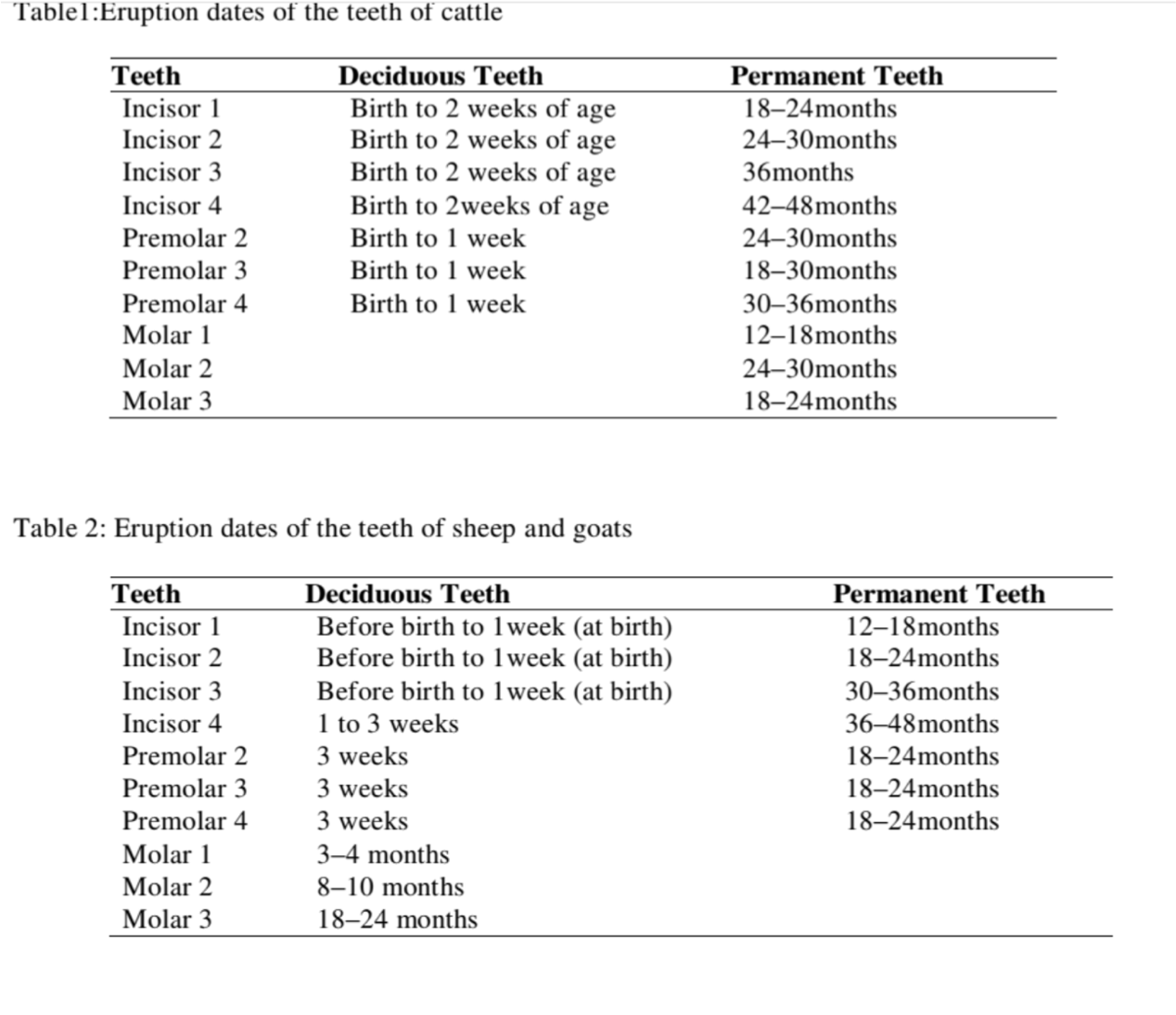

Supplement: Supplementary file 2 — Appendix 4 Field Sample Collection Reference Documents for Calf Enrolment [file VMS3-8-2157-s001.docx]
